# Cognition and Its Shaping Effect on Sexual Conflict: Integrating Biology and Psychology

**DOI:** 10.3389/fnint.2022.826304

**Published:** 2022-04-18

**Authors:** Beatriz Álvarez, Joris M. Koene

**Affiliations:** ^1^Department of Psychology, Universidad de Oviedo, Oviedo, Spain; ^2^Department of Ecology and Evolution, Amsterdam Institute for Life and Environment (A-LIFE), Vrije Universiteit, Amsterdam, Netherlands; ^3^Naturalis Biodiversity Centre, Leiden, Netherlands

**Keywords:** sexual conflict, cognitive processes, reproductive investment, ontogeny, classical conditioning

## Abstract

While genetic variation is of crucial importance for organisms to be able to adapt to their ever-changing environments over generations, cognitive processes can serve the same purpose by acting at shorter time scales. Cognition, and its resulting behaviour, allows animals to display flexible, fast and reversible responses that, without implying a genetic change, are crucial for adaptation and survival. In the research field on sexual conflict, where studies focus on male and female mating strategies that increase the individual’s reproductive fitness while forcing a cost on the partner, the role that cognition may play in how such strategies can be optimised has been widely overlooked. However, a careful analysis of behavioural studies shows that animals can develop and change their responses depending on what they perceive as well as on what they can predict from their experience, which can be of prime importance for optimising their reproductive fitness. As will be reviewed here, largely psychological processes, such as perception, memory, learning and decision-making, can not only modulate sexual conflict, but can also have a big impact on the reproductive success of a given individual. This review highlights the need for a more integrative view of sexual conflict where cognitive processes are also considered as a fundamental part of an animal’s adaptive mating response.

## Introduction

In the case of sexual reproduction, where both males and females are required for it to be successful, optimal fitness is generally not reached simultaneously by both sexes (Parker, 2006). This has been argued to be due to the differential investment in gamete production that is faced by males and females, as egg production is more costly than sperm production (e.g., Bateman, 1948; Schärer et al., 2012, but see also e.g., Dewsbury, 1982; Janicke et al., 2016). As a consequence of such initial differential investment, reproductive fitness for females is said to rely on their capacity to produce eggs, whereas for males it is argued to depend on their access to females (e.g., see Arnold, 1994 for a review). It is such differences in gamete production and subsequent interests on the two sides that are taken as the starting point of what is referred to as sexual conflict.

Before addressing the main topic—the modulating role of cognition on responses that are related to sexual conflict—we would like to briefly point out some of the problems with the definition of sexual conflict. On the one hand, sexual conflict has been defined as “a conflict between the evolutionary interests of individuals of the two sexes” (Parker, 1979, p. 124). However, as we have argued previously (Alvarez and Koene, 2018), we think we cannot state that males and females have evolutionary interests as such, and that it is more accurate to say that they have individual (albeit not necessarily conscious) interests, i.e., increasing one’s benefits with the lowest possible costs, with evolutionary consequences. Just to mention one extreme example, male seed beetles (Coleoptera: Bruchidae) possess spiny genitalia that allow them to anchor themselves to the female while mating (Stutt and Siva-Jothy, 2001) and to transfer proteins, *via* the wounds that are caused, that increase their male fertilisation success (Hotzy et al., 2012). This is beneficial for males as it helps them securing paternity, but the injuries inflicted by the spines to the female copulatory tract pose a high cost for the females, including a reduced longevity and reproductive success (Stutt and Siva-Jothy, 2001). The conflict arises because such strategies entail a cost for their partner and, as a consequence (and not as an aim), an evolutionary arms race can emerge between males and females as they try to adapt and to counter-adapt to the strategies employed by the other sex (e.g., Arnqvist and Rowe, 2005; Koene, 2012; Rice and Gavrilets, 2014; Alvarez and Koene, 2018). In the case of seed beetles, females have evolved thicker tracts so they can resist to the harm induced by their mating partner (Rönn et al., 2007).

On the other hand, if we stick to the definition of sexual conflict in terms of evolutionary interests, sexual conflict can then be seen as a cooperation between males and females to increase their net reproductive fitness in the long run instead of as a conflict (e.g., Cordero and Eberhard, 2003). For example, female seed beetles mating with males who are able to induce larger injuries (i.e., that further increase their paternity success) will sire sons with that same capacity, increasing the long-term fitness of that female, and males mating with females with thicker copulatory tracks will sire daughters with increased survival likelihood and thus also potentially increased maternal success. Thus, depending on the perspective that is taken, whether we should talk about conflict or collaboration to reach common goals becomes debatable. In fact, a clear and consensual definition of sexual conflict is still lacking (see Tregenza et al., 2006) and many studies on the topic do not provide a clear definition of sexual conflict.

Having all this in mind, and being aware that our definition may still need to be refined, we have defined sexual conflict, at an individual level, as the disagreement over investment that ensues because males and females adopt or develop strategies that are only aimed to increase their own fitness but that impose a cost on the mating partner (see Alvarez and Koene, 2018). As stated by Kokko and Jennions (2014), “sexual conflict can occur over every facet of breeding”. At the pre-copulatory level, it starts with the investment (or not) in searching for a mate, deciding to accept (or reject) a potential mate, the number of matings that take place and how many gametes are transferred. At the postcopulatory level, conflict can exist over additional matings (with the same or different individuals), induced physiological effects or physical harm, the number of and investment in offspring, and the amount of parental investment.

When we focus on the costs, i.e., what we consider the actual root of the conflict, that are faced by both males and females when mating, there is indeed a great variety of studies that provide clear evidence of both sexual conflict and of the sophistication of the mechanisms involved (Arnqvist and Rowe, 2005; Koene, 2012; Rice and Gavrilets, 2014; Alvarez and Koene, 2018). What is notable is that, regardless of the particular mechanism and species under study, most of the research conducted on sexual conflict has largely focussed on the physiological responses that are involved, as well as on the genetic variations underlying such physiological processes (Chapman et al., 2003; Rice and Gavrilets, 2014). For instance, on the male side, studies analyse how the production and transfer of seminal fluid proteins increase male fitness but decrease female fitness (see e.g., Poiani, 2006; Chapman, 2008; Koene, 2012; Perry et al., 2013). An example of this can be seen in fruit flies, where it has been shown that, among other effects, seminal fluid proteins increase females’ investment in egg production (Wigby and Chapman, 2005) and decrease their life expectancy because of toxic effects that these proteins have (Lung et al., 2002). An example from the female side are studies that reveal how (cryptic) female choice affect male fitness as determined by the degree of sperm acceptance (Firman et al., 2017). After all, such “sperm rejection” (*via* sperm ejection or sperm digestion), is a female driven process that is costly for the male since he has invested in the production of spermatozoa, accessory gland products, mate searching, courtship, and copulation, possibly after investing in competition against other males (see e.g., Dewsbury, 1982; Janicke et al., 2016).

However, as it has been increasingly pointed out by researchers of fields like that of evolutionary biology, physiological and genetic responses cannot be fully understood in the absence of the social environment and the cognitive processes that are constantly regulating animals’ activity (sensu organic selection by Baldwin, 1896 (see e.g., West-Eberhard, 2003; Diogo, 2017; for general reviews). Within the particular domain of mating, the modulating role of cognition has become increasingly acknowledged (e.g., Bateson and Healy, 2005; Prum, 2017; Ryan, 2021). Animals need to be able to identify sexual partners as such, to distinguish between receptive and non-receptive conspecifics, to identify their own sexual arousal, to assess the quality of a potential mate and to pursue him or her to achieve successful mating (e.g., Pfaus et al., 2001). Moreover, this evaluation needs to be orchestrated with the constant monitoring of the ever-changing environmental conditions of the given time and space in which mating is about to take place. In other words, mating is importantly affected by cognitive processes that include motivation, perception, learning, memory and decision-making (see Pfaus et al., 2001 for a review on how learning shapes mating in rats) and thus, they cannot be overlooked if we aim to understand it fully (see Bateson and Healy, 2005; Ryan, 2021 for a more general review). Likewise, as we have previously pointed out (Alvarez and Koene, 2018), we think that the current available data on sexual conflict cannot lead to a comprehensive understanding of this topic since it leaves out an important aspect of an animal’s life, i.e., its ontogeny, that, as will be further argued, can crucially shape the mating response and, therefore, the outcome of the sexual conflict (i.e., the costs) that is faced at each mating encounter. With the term ontogeny we refer to the individual’s experience and how this affects the development of behavioural patterns as well as the display of physiological responses over time. It is thus an adaptive response, to the particular scenario of mating, that is not accounted for by genetic aspects but by flexible and fast responses that are mediated by cognitive processes (Baldwin, 1896; West-Eberhard, 2003; Diogo, 2017).

In this regard, it is interesting to note that many authors do refer to essentially psychological processes such as anticipation, which is an animal’s prediction based on previously acquired knowledge, as determinants of reproductive investment (see e.g., Cattelan and Pilastro, 2018; Dore et al., 2018; Fuss, 2021), which is directly linked to sexual conflict (Kokko and Jennions, 2014). However, in the research field of sexual conflict, to our understanding, and to the best of our knowledge, a conceptual gap still exists, and we think it is limiting the way in which it is being analysed. Interestingly, although sexual conflict has been suggested as a source for cognitive variation (Cummings, 2018), the way in which cognitive processes may be affecting sexual conflict remains largely unexplored, and at both theoretical and experimental levels, the explanation of mating traits that are related to sexual conflict is mostly given in purely physiological or genetic terms (Arnqvist and Rowe, 2005; Rice and Gavrilets, 2014; Chapman, 2015). We think that the reason for not taking into account cognitive processes and their modulatory role on sexual conflict largely stems from an implicitly-assumed definition of mating as an automatic or innate response that is solely driven by physiological mechanisms that are in turn determined by genetic factors. This implicit theoretical framework, which seems to be shared by the majority of researchers, as inferred from the main reviews on the topic (Arnqvist and Rowe, 2005; Rice and Gavrilets, 2014; Chapman, 2015), leaves out cognitive processes that are fundamental for mating to occur and that, necessarily, have the potential to modulate sexual conflict.

We think that there is already evidence showing that cognitive processes exert a big influence on the way sexual conflict mechanisms work, but that they have not been analysed in such terms nor have they been conceptually taken together to widen the frame from which sexual conflict is understood. The aim of this review is therefore, without being exhaustive, to put together available examples that highlight the need for a more integrative view and approach. Most of the examples will highlight how different cognitive processes are intertwined with sexual conflict responses that allow males to increase their chances of fertilising eggs, compared to baseline conditions, which implies a higher investment on the female side, or that determine the degree of sperm acceptance by females (the less they accept, the higher the costs for the male; the more they accept, the higher the costs females may face). As a whole, they illustrate that cognition has a clear impact on sexual conflict and they show that individual ontogeny matters not only when it comes to mating (Pfaus et al., 2001) but also when talking about sexual conflict.

### Mate Choice and Sexual Conflict: Perception, Memory, Comparison, and Decision-Making

Mate choice could be regarded as more related to sexual selection than to sexual conflict, since preferences for a relatively better-quality partner or the rejection of a non-preferred partner constitute just an attempt to maximise one’s reproductive fitness. However, as argued by Kokko and Jennions (2014), whenever there is sexual selection, sexual conflict is also present, and mate choice has clear consequences in terms of sexual conflict. For example, both males and females have been found to invest significantly less in their own offspring if they were mated with a non-preferred partner, that they showed preference for in a choice test. Such smaller investment is costly for the partner because it can imply lower survival for the offspring, reducing overall reproductive fitness, and, as argued by Kokko and Jennions (2014), it can be seen as a conflict between the mating partners (over e.g., provisioning of the young). This can be observed in young mice, that have been shown to have a decreased survival rate when their fathers were mated with non-preferred, compared to preferred, females (Gowaty et al., 2003). Likewise, female canaries that have been exposed to unattractive male songs decrease the allocation of testosterone to their own eggs, which is known to compromise the survival rate of their own offspring (Gil et al., 2004). Mate choice is thus highly related to sexual conflict.

Although mate choice has been defined as a cognitive process that starts off with the perception and assessment of a conspecific (e.g., Bateson and Healy, 2005; Ryan et al., 2009; Dougherty, 2020; Ryan, 2021), as pointed out previously, it often seems to be understood as driven by an output from a nervous system that receives a specific amount of stimulation that triggers a sexually receptive response (i.e., a physiological response more than a cognitive one; e.g., Bakker, 1999; Iwasa and Pomiankowski, 1999; Andersson and Simmons, 2006; Kopp et al., 2018). It is true that, within any given species, sensory organs are responsive to specific ranges of stimulation and, as such, it is natural to observe predispositions for particular stimuli that are common to the majority of the individuals of the opposite sex (e.g., natural preference for red bellies in female guppies, Kodric-Brown, 1993; see also Ryan, 2021 for a review). However, this does not imply that perceptual preferences are purely innate nor fixed (e.g., see Weary et al., 1993). Experience with less or more similar phenotypes is important for subjects to learn to discriminate among them. For example, although pheromones can be seen as chemicals that trigger sexual arousal in a mechanistic way, studies in male rats have shown that these animals need to learn about them. Sexually naïve male rats do not display a preferential distinction between the odours from receptive or non-receptive females; even when they are experienced, they also need to learn not to attempt mating with a non-receptive female, despite the fact that she is not producing pheromones indicative of sexual receptivity (reviewed in Pfaus et al., 2001; see also e.g., Dukas, 2005 for another example in fruit flies). Just as experience (i.e., memory) can shape the response to chemicals, it can also shape perceptual preferences for certain phenotypes (see Witte and Nöbel, 2006 for a review). An example of this can be found in butterflies, where sexually naïve females prefer mating with wild-type males that have two eyespots on their wings rather than with males with what is called “enhanced ornamentation” (i.e., four eyespots), unless they had been exposed to the ornamented males before. In the latter case they showed a higher preference for enhanced ornamentation to the detriment of wild type males (Westerman et al., 2012).

Simple exposure to a phenotype does not only alter mate preferences, but also has clear consequences in terms of sexual conflict. For example, in the Pacific field cricket (*Teleogryllus oceanicus*), females that have experience with an unattractive male (defined by the type of songs he produces) will show a higher predisposition to mate with a second unattractive male and, what is crucial in terms of sexual conflict, to retain the spermatophore of the latter for longer (Rebar et al., 2011). In other words, mere exposure to a type of (unattractive) male results into acceptance of a higher amount of sperm from that type of male, increasing females’ costs, thus shaping sexual conflict. In wolf spiders (*Schizocosa uetzi*), the consequences of experience-based mate preferences can be even more drastic. In an experiment where the tibia of males’ forelegs was painted either brown or black, females that had never been exposed to any of the two types did not show any preference for either phenotype. On the contrary, if they had been exposed to just one of them before reaching maturity, not only did they show an increased likelihood to mate with the already known phenotype, but they also displayed an increased probability to cannibalise the male with the unfamiliar phenotype, clearly illustrating the conflict caused by sexual cannibalism (Hebets, 2003). As argued above, this situation could be understood just from the perspective of sexual selection (i.e., the female is minimising her reproductive costs as she is avoiding mating with an unpreferred male), but it is also an example of extreme sexual conflict as costs for the male reach maximum values.

More evidence showing that cognitive processes affect sexual conflict at the stage of mate choice comes from the fact that the attractiveness of a mate is not an absolute and that sexual conflict varies accordingly. In the aforementioned female guppies of the species *Poecilia reticulata*, mate choice is not solely regulated by the exact amount of colouration of the male partner (i.e., the objective physical qualities, determined by the presence of carotenoid -yellow, orange or red- skin spots). Indeed, females’ willingness to accept more or less sperm from a given male was shown to depend on the relative quality of that male: males of intermediate levels of attractiveness inseminated about three times the sperm (1521.3 × 10^3^ spermatozoa) when the female had to choose between them and a “less attractive” male than when the female had to choose between the same intermediate attractive male and a more attractive one (534.4 × 10^3^ spermatozoa) (Pilastro et al., 2004). Although we do not know the exact female physiological response that is involved (e.g., sperm digestion or sperm ejection), these results also show that females’ assessment of the attractiveness of one male relative to that of others, leads to a lower or higher cost for the males under assessment, since they invested in sperm, accessory gland products, courtship and copulation behaviour in each mating.

The rejection of sperm in each reproductive event, which implies a cost for the male, and thus, is a source of sexual conflict, would also be expected to change according to other variables that are known to affect mating. For example, in guppies, females alter their initial mate preferences after observing an older, but not a younger, female close to a male that they would not have chosen (Dugatkin and Godin, 1992; Godin et al., 2005). In these mate-choice–copy studies, the quantity of sperm that females accepted from males that were preferred or non-preferred by older or younger females was not quantified. Thus, we cannot know to what extent it may affect sexual conflict. However, there is evidence coming from the feral fowl (*Gallus gallus*) showing that the assessment of the social environment in a particular temporal moment is also shaping sexual conflict. In the feral fowl, females have been observed to eject, at least, half the sperm of non-dominant males but not that of dominant males. Importantly, the rejection of ejaculates can be altered when the hierarchy is changed after removing dominant and subdominant males (Pizzari and Birkhead, 2000). In both species, sperm acceptance/rejection depends on the notion of relative quality, which importantly implies not only perceiving but also comparing the different available options and deciding the optimal response to each situation. Whether females accept less, more or none of the sperm will depend on the outcome of those cognitive processes and so will the costs faced by the males, in terms of sperm and accessory gland proteins production, courtship and copulation in each mating encounter.

The examples just mentioned are instances of how the cognitive processes that affect sexual selection necessarily also affect sexual conflict. Although the boundaries between sexual selection and sexual conflict could be argued not to be clear enough in some of them (see Kokko and Jennions, 2014; see also Arnqvist, 2004 for a discussion on the difficulty of establishing boundaries between the two concepts), we use these examples to illustrate that, with time, individuals accrue knowledge about their environment, including possible sexual partners (and possibly also the costs that mating with these entail), through (direct or not) experience, and that this knowledge determines the willingness to mate with, i.e., to accept/reject more or less sperm from, a particular partner. In that sense, we can say that cognitive processes regulate the activation of physiological or behavioural responses that are central to sexual conflict such as those related to sperm acceptance or to resource allocation to the offspring. It is the constant execution of such cognitive processes that allows, both males and females, to assess the quality of their partner and therefore, to flexibly adjust their response to the particular situation. Thus, cognitive processes such as perception, memory, comparison and decision-making can be said to play a modulatory role in sexual conflict that affects mating at all stages, from mate recognition to mate acceptance or to offspring resource allocation.

### Preparedness: A Special Type of Learning Experience

As stated above, anticipation to a mating encounter has been argued to be a determinant of reproductive investment. As a matter of fact, being able to foresee a mating encounter allows animals to adopt different strategies to the specific scenario where mating is about to take place (see Hollis, 1982 for a discussion on the concept of preparedness). It is thus of great interest for animals to learn to identify and to pay attention to specific cues that signal the availability or receptivity of a potential partner so that the likelihood of being accepted increases, which in turn affects the level of sexual conflict.

A special type of learning experience that allows animals to anticipate relevant events is that of classical conditioning (also referred to as Pavlovian conditioning). Classical conditioning occurs when an initially neutral stimulus (e.g., an acoustic stimulus such as the sound of a metronome) is presented together with another stimulus of biological relevance, such as food, that is referred to as the unconditioned stimulus (US). After repeated presentations, the neutral stimulus becomes a conditioned one (CS) that signals the presence of the US, and animals typically increase their rate of responding to the CS without needing to wait for the US to be present (Pavlov, 1927/2003).

Within the reproduction scenario, classical conditioning is important for the development of a correct mating response since it helps animals to learn about specific cues (CSs) that are consistently paired with successful mating (US). An already cited example is that of male rats (see Pfaus et al., 2001 for a review on the role of learning in rats’ mating responses), that show an increased preference for odours (CSs) that are indicative of female sexual receptivity because they have been associated with successful mating (US). Indeed, Pavlovian conditioning has been shown to be of great importance for the development of preferences for pheromones, for the correct discrimination between receptive and non-receptive females and for a conditioned ejaculatory preference for certain females (see Pfaus et al., 2001 for rats; see also e.g., Dukas, 2005 for fruit flies or Domjan and Gutiérrez, 2019 for Japanese quail).

Classical conditioning is, thus, crucial for the successful identification of a sexual partner that leads to successful mating. Importantly, accumulated successful mating experiences determine physiological responses that are involved in sexual conflict mechanisms. For example, in male rats, the more sexual experience they gain, the larger testes, the heavier penises and the greater the secretions from male accessory glands (reviewed in Pfaus et al., 2001). As already mentioned, these gland secretions are one of the most widespread sexual conflict mechanisms that induce high costs for females such as a decrease in sexual receptivity, an increase in egg investment, or even a decrease in their survival rate (see Poiani, 2006; Koene, 2012; for reviews on the costs of accessory sex gland secretions; see also Ramm and Stockley, 2016 for a review in rodents). Such a link between classical conditioning, more mating experience and increased male accessory gland products, indicates that classical conditioning can play a modulating role in sexual conflict.

Pavlovian conditioning has been shown to be a useful tool for animals of different species to increase their reproductive success. For example, experiments with blue gourami (*Trichogaster trichopterus*), in which males were exposed to a light (CS) followed by the presence of a female, showed that being able to use the light as a predictor for the arrival of a female resulted in less aggressive behaviour toward her (see Hollis, 1999 for a review of the different studies she led on associative learning and mating in blue gourami). Such behavioural change in the male induced the females to spawn faster than the females that were being courted by males who had not been subjected to that learning experience. More importantly, the number of offspring sired by classically conditioned males was significantly higher than that sired by the control males for whom the CS had been unreliably paired with the presence of the female. When looking at the results obtained in this experiment (Hollis et al., 1997), the difference in the number of offspring produced by conditioned and by control males is in the magnitude of hundreds of fry. If we take the control group, i.e., females who mated to males that were not able to anticipate their availability, as the baseline of females’ investment, assuming that males were of equal quality across groups, we can say that, in this particular scenario, classical conditioning prepared males for the mating encounter in such a way that they increased their reproductive fitness by inducing females to increase their investment, i.e., the costs, by producing or releasing a significantly higher number of ova.

Another example of how classical conditioning induces the sexual partner to increase its reproductive investment is observed in the Japanese quail *Coturnix japonica.* When males are exposed to cues that signal a mating encounter, they produce larger ejaculates and larger numbers of spermatozoa (Domjan et al., 1998). With a differential conditioning procedure, in which males were exposed to a female in one context but not in a different one, mating in the context that had been paired with access to a female increased males’ mating success, as measured by the number of fertilised eggs (78 vs. 39%; Adkins-Regan and MacKillop, 2003). It is important to note that the females used during training were different to the ones used in the test (i.e., higher fertilisation rate cannot be explained by a higher male preference for a particular female); that the females used in the test were sexually naïve, and that they had been randomly assigned to mate in either one context or the other (i.e., female preferences for males was not taken into account, so this variable can either explain the differences observed in the number of fertilised eggs). Thus, males that were able to rely on external cues were also able to impose a higher reproductive cost to the female they mated with.

This ability to anticipate a mating encounter was shown to be of special importance in situations where one female mated with two different males. When this occurs, paternity is usually shared equally between the two males (i.e., 50-50%). However, when two males are competing for a single female and one of them has been subjected to Pavlovian conditioning, the one who is able to rely on a cue that signals the availability of the female is quicker at mating and, most relevant, sires a higher proportion of the offspring (72 vs. 28%). Such higher paternity success was shown to be explained by that learning experience alone, independent from whether they mated first or second with the female (Matthews et al., 2007). As argued by the authors, males of both conditions had the same mating experience during the experiment and prior to the test, so differences in fertilisation were not likely to be due to differences in sperm production but in sperm release, and/or, as argued by Adkins-Regan and MacKillop (2003), the higher paternity rates could also be due to higher production of foam (a substance that is transferred to the female during copulation and that is known to increase fertilisation success). Although research on the exact physiological mating response affected by classical conditioning is still lacking, these results show that classical conditioning allowed males to increase females’ investment toward their own sperm, from the baseline of 50%.

The experiments conducted with blue gourami and Japanese quail show that using cues as indicators of a mating opportunity helps individuals to prepare better for the mating encounter but that much research still needs to be done to analyse the effects of classical conditioning in terms of sexual conflict. Nonetheless, they also show that classical conditioning is a special instance of learning with a potentially important modulatory role in sexual conflict: males who can predict the availability of a (fertile) female adjust their mating response to obtain a higher reproductive fitness, independently of female preferences (so to the female’s costs), by optimising the mating process in terms of copulation time and/or amount of sperm transfer, and possibly also by modulating the amount of accessory gland secretions that are transferred along with sperm (as happens e.g., in *D. melanogaster*; Mohorianu et al., 2018).

### A Yet To-Be-Developed Experimental and Theoretical Field

As pointed out above, studies on the cognitive processes that shape mating responses and on the physiological mechanisms that are involved in sexual conflict are largely performed independently from one another. The evidence here gathered shows that, just as cognitive processes play an important role in sexual selection, there exists a modulatory relationship between cognition and sexual conflict that has not been fully explored and we are convinced that properly considering the interplay between cognitive processes and physiological mechanisms in the context of reproduction will be a fruitful direction for this field of research.

For example, as pointed out earlier, studies in guppies have not measured the extent to which social variables that are already known to affect the attractiveness of a potential mate alter the amount of sperm that a female is willing to accept, which would allow to measure the cost suffered by the male. Likewise, the studies with blue gourami and Japanese quails also reveal that much research needs to be done to understand the modulatory effect of classical conditioning. The experiments reported show beyond doubt that Pavlovian conditioning can serve to increase the partner’s investment (as measured e.g., by the larger number of eggs laid by blue gourami females), but they also show that learning results in an increased investment by the individual that is able to anticipate the mating encounter (as shown by the production of larger ejaculates by learning Japanese male quails). The extent to which the extra gains obtained from being able to predict a mating encounter outweigh the costs remains to be explored.

Just like learning males, females also increase their own investment when they can predict a mating encounter. In an experiment conducted with Japanese quail, it was observed that sexually naïve females who had been subjected to Pavlovian conditioning in which a context was paired with just the presence of a male (copulation was prevented) laid a higher proportion of fertilised eggs when they mated in the context that had been consistently paired with the presence of males, compared to the females that mated in a context in which males were never present (Adkins-Regan and MacKillop, 2003). In this particular situation, the males that the females encountered at the test were novel to them, as they had not been exposed to them during training. Just knowing that the cage in which they were introduced was a good predictor for the presence of a male was enough to affect females’ reproductive success, also increasing the reproductive benefits of the male. The physiological responses that were modulated by this learning are still unknown. Pavlovian conditioning may alter sperm rejection or it might also affect the composition of the female reproductive tract fluid, leading, in this particular case, to increased chances of successful insemination and lower costs for the male. In this regard, female reproductive tract fluid is much understudied, but there is very recent evidence showing that its composition changes after mating (McDonough-Goldstein et al., 2021). It would thus be interesting to test whether Pavlovian conditioning could also alter the composition of females’ fluid, just as it changes seminal fluid in males.

Finally, when taken together, most of the studies seem to indicate that experience with a given partner or phenotype enhances animals’ motivation to mate, thus it seems that learning is mostly playing a facilitating role. However, we prefer using the term “modulatory” instead of facilitating because it might also serve to hamper mating under specific circumstances. For example, in mosquitofish (*Gambusia holbrooki*), where coercive mating occurs, it has been observed that male sexual harassment results in a decreased foraging efficiency, and that females can reduce such costs by aggregating with other females (Pilastro et al., 2003; Dadda et al., 2005). It could thus be possible that if, after classical conditioning training, females are able to anticipate an unwanted mating encounter, they might be able to avoid mating entirely or, if not possible, to display sexually antagonistic strategies to a maximum level.

## Discussion

As we have argued, the research that has been reviewed here shows that sexual conflict strategies are not solely determined by genetic or physiological responses that are independent of the knowledge that animals acquire throughout their life. On the contrary, it shows that experience, i.e., ontogeny, affects the way in which those sexual conflict strategies are displayed, altering the amount of reproductive investment a mating partner will face. Experience, whether interpreted in terms of Pavlovian conditioning or not, affects the mating sequence and the way in which sexual conflict responses are displayed. These responses range from motivation to mate to the number of resources that are allocated to the offspring sired by a particular partner.

Genetic variability is without question a driving force for evolution. The differences in both the genotype and phenotype provide a source of trait variability that enables organisms to adapt to the surrounding conditions of their environment. On the one hand, these adaptations take considerable (evolutionary) time to occur, which would render an individual in a vulnerable state: if the genetic variation has not been inherited or no beneficial mutation has taken place, there is little room for the organism to adapt successfully. On the other hand, cognitive processes are constantly ongoing and allow animals to monitor their surroundings as well as their internal states, to accumulate and update their knowledge about their *Umwelt* and to regulate and/or adjust their behaviour accordingly. As a consequence, cognitive processes and behaviour offer temporal, flexible and quick responses that are shorter- or longer-term adaptations that can be as crucial for survival as evolutionary changes. Indeed, learning and behaviour have been argued to be one of the main driving forces of evolution (Baldwin, 1896; Roe and Simpson, 1958; Piaget, 1976; West-Eberhard, 2003; Ginsburg and Jablonka, 2010; Diogo, 2017) because they allow animals to successfully regulate their activities on a daily basis. Here it is important to highlight that although cognitive abilities (e.g., attention, memory span, processing speed or the ability to learn) may become common, and variable, due to genetic variations (Plomin et al., 2013, but see also Nisbett et al., 2012), the adaptive response an animal develops to a particular situation is not genetically determined. In that sense, the acquired behavioural response can be reversible as it can change according to e.g., learning contingencies (e.g., counterconditioning), and most importantly, it does not involve a genetic change that is inherited by the offspring. In that regard, cognitive processes have been considered as sources of new and fast adaptations in research areas such as that of comparative psychology (Shettleworth, 2009) or eco-evolutionary dynamics (Svensson, 2019), but they have been overlooked in the field of sexual conflict (e.g., Chapman et al., 2003; Shackelford and Goetz, 2012). As we have already discussed, most of the sexual conflict strategies that animals employ are still largely examined under a very mechanistic approach in which only physiological mechanisms and genetic traits seem to be considered (Alvarez and Koene, 2018). The current review shows that there is still a conceptual gap that needs to be addressed in the domain of sexual conflict as there is ample evidence that sexual conflict is not solely regulated by genetic traits but also by each individuals’ assessment of different aspects within a mating situation. Experience affects the way in which a mating partner is perceived, the motivation to mate or the capability to prepare for a mating encounter altering the physiological response that will be displayed.

As pointed out by Tinbergen (1963), we cannot reach a comprehensive view of any biological trait without taking into consideration both ultimate and proximal causes. Importantly, ontogeny of behaviour cannot be understood as directly determined by genetic factors (reviewed by Sánchez and Loredo, 2007), but rather as the result of cognitive processes (motivation, attention, and perception), experience (learning and memory) and the subsequent decisions. Cognitive processes are fundamental for displaying an adaptive mating response and they modulate the physiological responses an animal experiences in a particular moment, including those related to sexual conflict. The fact that ejaculate size, sperm acceptance or paternity success are enhanced under certain cognitive conditions is already highlighting the need to take ontogeny into account for a good understanding of sexual conflict. We sincerely hope that this review will instigate a field of research that will focus on the interplay of the two factors, closing the existing gap.

## Author Contributions

BÁ and JK: conceptualization. BÁ: writing—original draft. JK: co-writing original draft, and writing—review and editing. Both authors contributed to the article and approved the submitted version.

## Conflict of Interest

The authors declare that the research was conducted in the absence of any commercial or financial relationships that could be construed as a potential conflict of interest.

## Publisher’s Note

All claims expressed in this article are solely those of the authors and do not necessarily represent those of their affiliated organizations, or those of the publisher, the editors and the reviewers. Any product that may be evaluated in this article, or claim that may be made by its manufacturer, is not guaranteed or endorsed by the publisher.
